# Evaluation of three stone-scoring systems for predicting SFR and complications after percutaneous nephrolithotomy: a systematic review and meta-analysis

**DOI:** 10.1186/s12894-019-0488-y

**Published:** 2019-07-01

**Authors:** Kehua Jiang, Fa Sun, Jianguo Zhu, Guangheng Luo, Peng Zhang, Yong Ban, Gang Shan, Changxiang Liu

**Affiliations:** 1 Department of Urology, Guizhou Provincial People’s Hospital, Guiyang, China; 2 Department of Urology, The Affiliated Hospital of Guizhou medical University, Guiyang, China

**Keywords:** Guy’s score, S.T.O.N.E. score, CROES nomogram, Stone free rate, Meta-analysis

## Abstract

**Background:**

Clinical studies assessing the feasibility and accuracy of three stone scoring systems’s (SSSs: Guy’s stone score, CROES nomogram and S.T.O.N.E nephrolithometry scoring system) have reported contradictory outcomes. This systematic evaluation was performed to obtain comprehensive evidence with regard to the feasibility and accuracy of three SSSs.

**Methods:**

A systematic search of Embase, Pubmed, Medline, and the Cochrane Library was conducted to identify studies that compared three SSSs up to Mar 2018. Patients were categorized according to stone free (SF) and no-stone free (NSF), Outcomes of interest included perioperative variables, stone-free rate (SFR), and complications.

**Results:**

Ten studies estimating three SSSs were included for meta-analysis. The results showed that SF patients had a significantly lower proportion of male (OR = 1.48, *P* = 0.0007), lower stone burden (WMD = -504.28, *P* < 0.0001), fewer No of involved calyces (OR = -1.23, *P* = 0.0007) and lower proportion of staghorn stone (OR = 0.33, *P* < 0.0001). Moreover, SF patients had significantly lower score of Guy score (WMD = -0.64, *P* < 0.0001), but, S.T.O.N.E. score (WMD = -1.23, *P* < 0.0001) and a higher score of CROES nomogram (WMD = 29.48, *P* = 0.003). However, the comparison of area under curves (AUC) of predicting SFR indicated that there was no remarkable difference between three SSSs. Nonetheless, Guy score was the only stone scoring system that predicted complications after PCNL (WMD = -0.29, 95% CI: − 0.57 to − 0.02, *P* = 0.03).

**Conclusions:**

Our meta-analysis indicated that the three SSSs were equally, feasible and accurate for predicting SFR after PCNL. However, Guy score was the only stone scoring system that predicted complications after PCNL.

**Electronic supplementary material:**

The online version of this article (10.1186/s12894-019-0488-y) contains supplementary material, which is available to authorized users.

## Background

The recommended treatment option for renal calculi and staghorn calculi is percutaneous nephrolithotomy (PCNL) according to the guidelines of the European Association of Urology (EAU) [1]. PCNL has increasingly been used over the past few decades and may continue in the future [2, 3]. However, PCNL outcomes among the authors are different, because of the vast heterogeneity in the methods for clinical and academic characterization of nephrolithiasis besides the evaluation of surgical outcomes. So assessing the preoperative factors that affect SFR and complications is critical.

The Guy’s stone score, the Clinical Research Office of the Endourological Society(CROES) nomogram and the S.T.O.N.E.(stone size, tract length, obstruction, number of involved calices and essence) stone score are seen as predictors of stone-free status (SFS) and complications after PCNL [4–6]. The widespread use of a standardized stone scoring system is very precious for counseling patient, clinical decision, and assessment of outcomes, in addition to improving academic reporting [7]. However, no universally accepted stone scoring system for predicting SFR and complications after PCNL exists. Comparison of the SSSs in different clinical studies indicated some advantages as well as disadvantages of one nomogram to another for different variables. Hence, we performed a systematic review of the literature with a meta-analysis of the available published literature to compare the feasibility and accuracy of three SSSs in predicting PCNL outcomes concerning SFR and complications.

## Methods

### Study selection

According to the Cochrane Handbook recommendations, a systematic review of published literature was performed [8]. To identify all studies published up to Dec 31, 2018, which assessed the feasibility and accuracy of three SSSs. The following MESH search headings were used: “comparative studies”, “Guy”, “CROES”, “S.T.O.N.E”, “percutaneous nephrolithotomy”, “stone free rate”, and “complication”.

### Inclusion and exclusion criteria

All studies included in this meta-analysis satisfied the following requirements: (a) compare the two or three SSSs, (b) report the outcomes of two or three SSSs, (c) document the surgery as PCNL, (d) document indications for PCNL with renal stones. Studies were excluded if: (a) the article did not meet the inclusion criteria, (b) no outcomes were mentioned or the parameters were impossible to analyze the three SSSs from the published findings.

### Data extraction and outcomes of interest

Two of the authors (JKH and SF) extracted data from the included studies including: author identification, country, publication years, study design, age, and the number of patients. All disagreements about eligibility were resolved by consensus through author discussions. The outcomes, including SFR and, overall complications, were extracted to compare between three SSSs. Overall complications were graded based on the Clavien-Dindo system [9].

### Study quality assessment

In accordance with the criteria of Centre for Evidence-Based Medicine in Oxford, we evaluated the level of evidence (LOE) of the included ten studies. Furthermore, Jaded Score was applied to evaluate the methodological quality of RCTs [10]. while the Newcastle-Ottawa Scale (NOS) assessed the methodological quality of non-RCTs observational studies [11]. Besides, JKH and ZJG evaluated the quality of the articles and discrepancies were rechecked and resolved by discussion.

### Statistical analysis

All analyses were conducted by Review Manager 5.3 (Cochrane Collaboration, Oxford, UK). Continuous and dichotomous variables were analyzed by weighted mean differences (WMDs) and odds ratios (ORs). All analysis results were reported with 95% CIs. *I*^2^ and *X*^2^ statistics were applied to evaluate the quantity of heterogeneity, and when I^2^>50%, the evidence was considered to have substantial heterogeneity, the random-effects (RE) model would be applied, otherwise, the fixed effects (FE) model was applied. Egger’s test and funnel plot evaluated the publication bias. Sensitivity analyses estimated the influence of studies with a high risk of bias on the overall effect.

## Results

### Characteristics of eligible studies

**Fig. 1:**
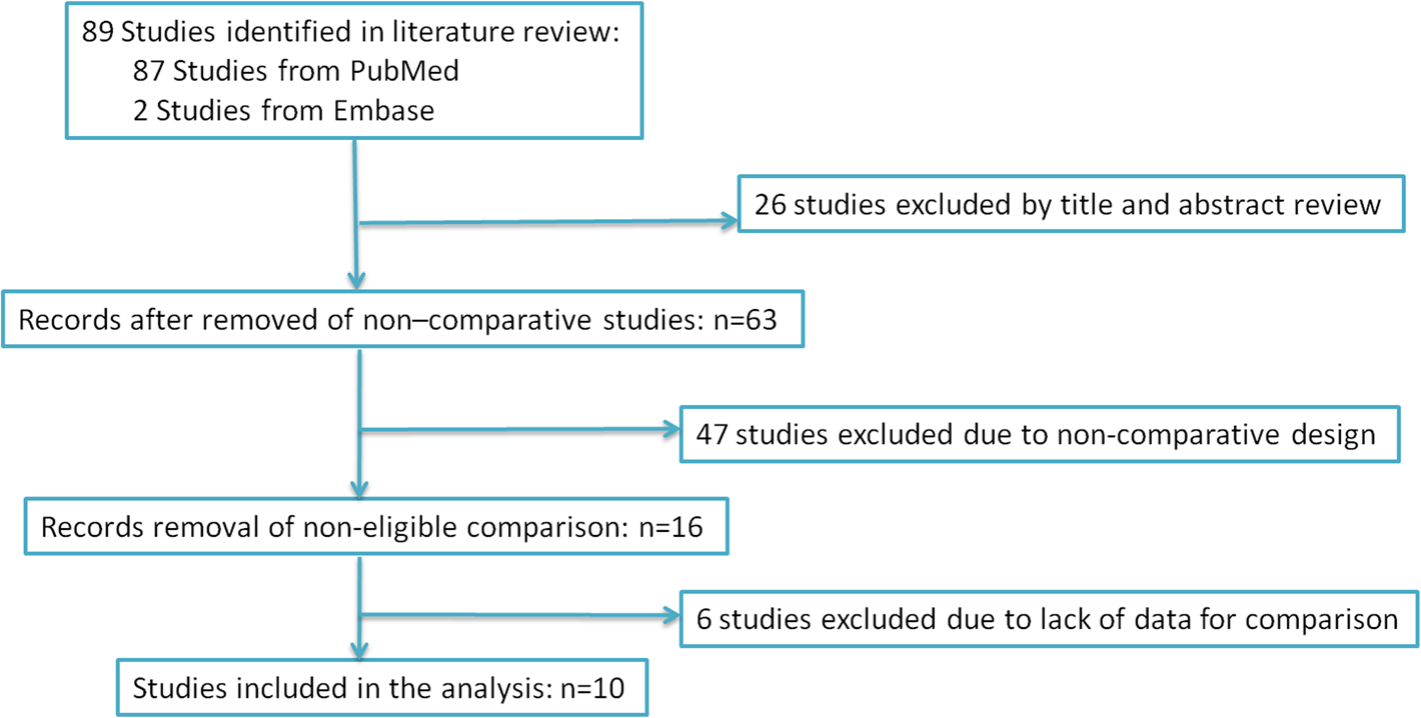
PRISMA diagram. The search strategy and number of studies identified for inclusion in this meta-analysis

Ten studies [12–21] conformed to the inclusion criteria of this meta-analysis and were there included in the analysis of three SSSs (Fig. 1). The demographic and clinical characteristics of the included literature were shown in Table 1.

**Table 1:** Characteristics of included studies

First author year	Country	Study interval	Design	LOE	No.of patients	Matching/comparable^a^
Bozkurt, 2015 [12]	Turekey	2012–2015	Retrospective	3b	437	1,2,3,4,5
Choi, 2016 [13]	Korea	2003–2014	Retrospective	3b	217	1,2,3,4,5,6
Choi, 2016 [14]	Korea	2012–2015	Retrospective	3b	141	1,2,3,4, 5,6
Jaipuria, 2016 [15]	India	2014–2015	prospective	3b	606	1,3
Kocaaslan, 2016 [16]	Turekey	2010–2015	Retrospective	3b	137	1,2,3,4, 5,6
Labadie, 2015 [17]	USA	2009–2012	Retrospective	3b	246	1,2,3,4, 5,6
Noureldin, 2015 [18]	Canada	2009–2013	Retrospective	3b	185	1,2,3,5
Sfoungaristos, 2016 [19]	Israel	2010–2015	Retrospective	3b	73	1,3,4, 5,6
Tailly, 2016 [20]	Canada	2006–2013	Retrospective	3b	586	1,2,3,4, 5,6
Yarimoglu, 2016 [21]	Turekey	2012–2015	Retrospective	3b	262	1,2,3,4, 5

^a^ Matching/comparable variable: 1 = age, 2 = BMI, 3 = gender, 4 = laterality(right/left), 5 = stone burden, 6 = stone density

### Quality of the studies and level of evidence(Table 1)

In this meta-analysis, the NOS quality assessment method of the observational studies [11], and the US Preventive Services Task Force grading system were applied to evaluate the quality of all studies [10]. Included studies were all level 3b. Also, the demographic variables of the three SSSs were extracted from included articles (Table 1).

### Description of included studies and preoperative characteristics of the patients (Table 2)

**Fig. 2:**
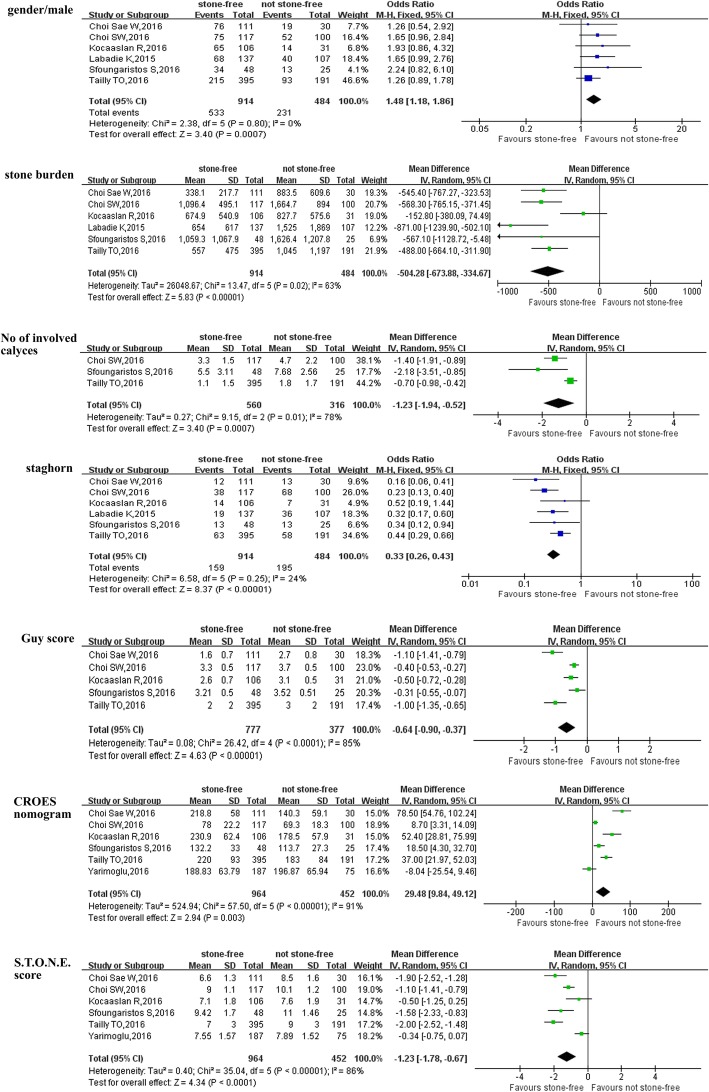
Forest plot and meta-analysis of preoperative characteristics between the stone-free patients and not stone-free patients

Patients were categorized as stone free(SF) and no-stone free(NSF), and the pooled data of included studies [13, 14, 16, 17, 19, 20] showed that stone free patients had significantly lower proportion of male(OR = 1.48, 95% CI: 1.18 to 1.86, *P* = 0.0007) (Fig. 2), lower stone burden (WMD = -504.28, 95% CI: − 673.88 to − 334.67, *P* < 0.0001) (Fig. 2), fewer No of involved calyces (OR = -1.23, 95% CI: − 1.94 to − 0.52, *P* = 0.0007) (Fig. 2) and lower proportion of staghorn stone (OR = 0.33, 95% CI: 0.26 to 0.43, *P* < 0.0001) (Fig. 2). And stone free patients had significantly lower score of Guy score (WMD = -0.64, 95% CI: − 0.90 to − 0.37, *P* < 0.0001) (Fig. 2), lower score of S.T.O.N.E. score (WMD = -1.23, 95% CI: − 1.78 to − 0.67, *P* < 0.0001) (Fig. 2) and higher score of CROES nomogram (WMD = 29.48, 95% CI: 9.84 to 49.12, *P* = 0.003) (Fig. 2) (Table 2). However, there were no remarkable difference in terms of age, BMI, laterality and stone density between SF and NSF patients (Table 2, Additional file 1: Figure S1).

**Table 2:** Overall analysis of demographic and clinical characteristics compared stone-free with not stone-free after PCNL

Outcomes of interest	No. of studies	No. of patients SF/NSF	OR/WMD(95% CI) ^a^	*p*-value	Study heterogeneity			
					Chi2	df	I^2^	*p*-value
Age(year)	7	1101/559	0.02[−1.48,1.52] ^a^	0.98	5.57	6	0%	0.47
BMI(kg/m^2^)	6	1053/534	−0.59[− 1.80,0.63] ^a^	0.34	21.87	5	77%	**0.0006**
Proportion/male	6	914/484	1.48[1.18,1.86]	**0.0007**	2.38	5	0%	0.80
Laterality(right/left)	6	914/484	1.10[0.88,1.38]	0.41	8.43	5	41%	0.13
Stone burden(mm2)	6	914/484	−504.28[−673.88,-334.67] ^a^	**< 0.0001**	13.47	5	63%	**0.02**
No of involved calyces	3	560/316	−1.23[−1.94,-0.52]	**0.0007**	9.15	2	78%	**0.01**
Staghorn	6	914/484	0.33[0.26,0.43]	**< 0.0001**	6.58	5	24%	0.25
Stone density(HU)	4	777/377	−37.65[−81.95,6.65] ^a^	0.10	4.90	4	18%	0.30
Guy score	5	777/377	−0.64[−0.90,-0.37] ^a^	**< 0.0001**	26.42	4	85%	**< 0.0001**
CROES nomogram	6	964/452	29.48[9.84,49.12] ^a^	**0.003**	57.50	5	91%	**< 0.0001**
S.T.O.N.E. score	6	964/452	−1.23[−1.78,-0.67] ^a^	**< 0.0001**	35.04	5	86%	**< 0.0001**

*SF* stone free, *NSF* not stone free, *PCNL* percutaneous nephrolithotomy, *OR* odds ratio, *WMD* weighted mean difference, *CI* confidence interval, *BMI* body mass index, ^a^ WMD

### Outcomes of perioperative variables (Table 3)

**Fig. 3:**
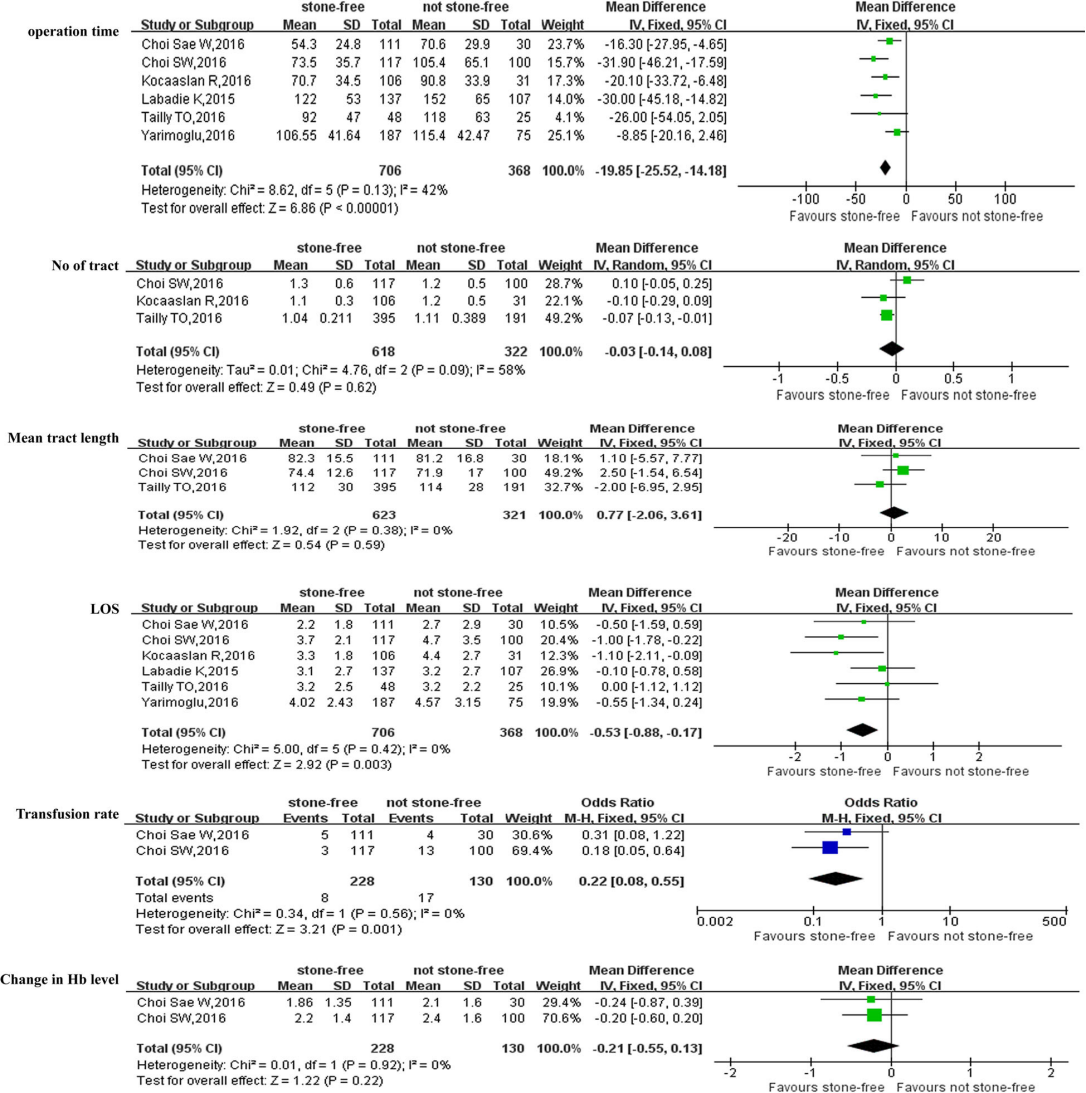
Forest plot and meta-analysis of outcomes of perioperative variables between stone-free patients and not stone-free patients

With respect to perioperative variables, pooled data of 6 studies [13, 14, 16, 17, 20, 21] involving 1074 participants demonstrated that SF patients were associated with shorter operative time than NSF patients (WMD:-19.85 min; 95% CI: − 25.52 to − 14.18; *P*<0.0001)(Fig. 3). SF patients were also associated with shorter length of hospital stay (LOS) (WMD: − 0.53 days; 95% CI: − 0.88 to − 0.17; *P* = 0.003) (Fig. 3) and lower transfusion rate (OR: 0.22; 95% CI: 0.08 to 0.55; *P* = 0.001) (Fig. 3) than NSF patients, respectively. However, there were no statistically difference between SF and NSF patients in term of No of tract (OR = -0.03; 95% CI: − 0.14 to 0.08; *P* = 0.62) (Fig. 3), mean tract length (WMD = 0.77; 95% CI: − 2.06 to 3.61; *P* = 0.59) (Fig. 3) and change in Hb level (WMD = − 0.21 g/mL; 95% CI: − 0.55 to 0.13; *P* = 0.22) (Fig. 3)(Table 3).

**Table 3:** Overall analysis of perioperative outcomes comparing stone free with not stone free after PCNL

Outcome of interest	No. of studies	No.of patients SF/NSF	OR/WMD(95%CI) ^a^	*p*-value	Study heterogeneity			
					Chi^2^	df	*I* ^*2*^	*p*-value
Operative time, min	6	706/368	−19.85[−25.52,-14.18]	**<0.0001**	8.62	5	42%	0.13
No of tract	3	618/322	−0.03[−0.14,0.08] ^a^	0.62	4.76	2	58%	0.09
Mean tract length	3	623/321	0.77[−2.06,3.61]	0.59	1.92	2	0%	0.38
LOS, days	6	706/368	−0.53[−0.88,-0.17]	**0.003**	5.00	5	0%	0.42
Transfusion rate	2	228/130	0.22[0.08,0.55] ^a^	**0.001**	0.34	1	0%	0.56
Change in Hb level(g/mL)	2	228/130	−0.21[−0.55,0.13]	0.22	0.01	1	0%	0.92

*NS* stone free, *NSF* not stone free, *LOS* length of stay, *PCNL* percutaneous nephrolithotomy, *OR* odds ratio, *WMD* weighted mean difference, *CI* confidence interval, ^a^ OR

### Outcomes of three scoring systems for predicting SFR after PCNL (Table 4)

**Fig. 4:**
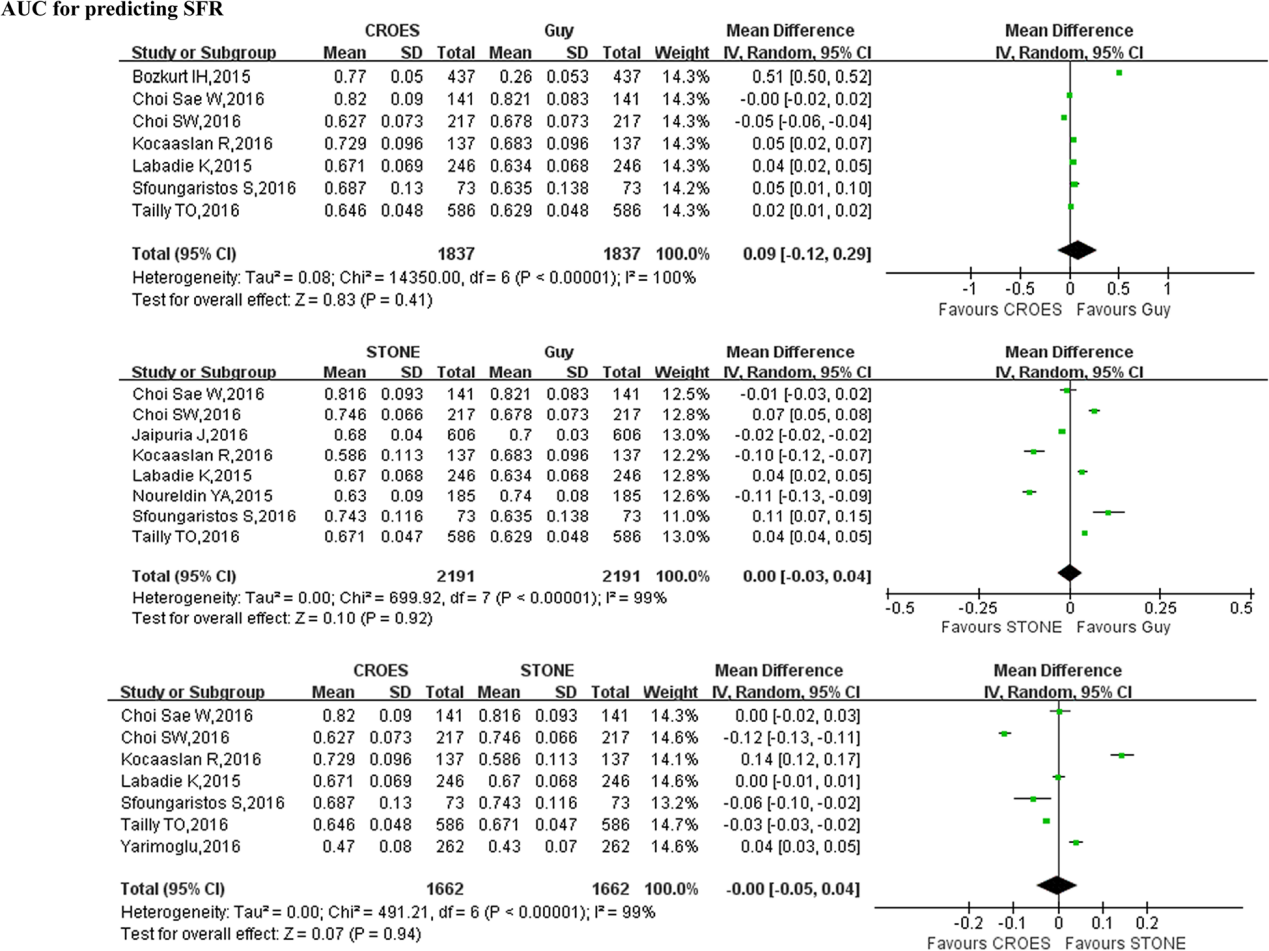
Forest plot and meta-analysis of outcomes of three scoring systems for predicting SFR after PCNL. SFR = stone free rate

Pooled data of 7 studies [5, 12–14, 16, 17, 20] reported an accuracy of three SSSs in predicting post-PCNL SFR, the forest plot indicated that there was no statistical difference between Guy score and CROES nomogram with respect to area under curves(AUC) of prediction of SFR(WMD = 0.09; 95% CI:-0.12 to 0.29; *P* = 0.41) (Table 4, Fig. 4). There was also no significant difference between S.T.O.N.E. score and Guy score with respect to area under curves (AUC) of prediction of SFR (WMD = 0.00; 95% CI:-0.03 to 0.04; *P* = 0.92) (Table 4, Fig. 4) and no significant difference between CROES nomogram and S.T.O.N.E. score with respect to area under curves (AUC) of prediction of SFR (WMD = 0.00; 95% CI: − 0.05 to 0.04; *P* = 0.94) (Table 4, Fig. 4).

**Table 4:** Overall analysis of three stone-scoring systems for predicting SFR after PCNL

Outcome of interest	No.of studies	No. of patients	MD (95%CI)	*p*-value	Study heterogeneity			
					Chi^2^	df	*I* ^*2*^	*p*-value
		**CROES/Guy**						
AUC	7	1837/1837	0.09[− 0.12,0.29]	0.41	14,350.0	6	100%	**<0.0001**
		**STONE/Guy**						
AUC	8	2191/2191	0.00[−0.03,0.04]	0.92	699.92	7	99%	**<0.0001**
		**CROES/STONE**						
AUC	7	1662/1662	−0.00[−0.05,0.04]	0.94	491.21	6	99%	**<0.0001**

*SFR* stone free rate, *PCNL* percutaneous nephrolithotomy, *AUC* area under curve, *CROES* clinical research office of the endourological society scoring system, *Guy* Guy scoring system, *S.T.O.N.E* S.T.O.N.E scoring system, *MD* mean difference, *CI* confidence interval

### Outcomes of three scoring systems for predicting complications after PCNL (Table 5)

**Fig. 5:**
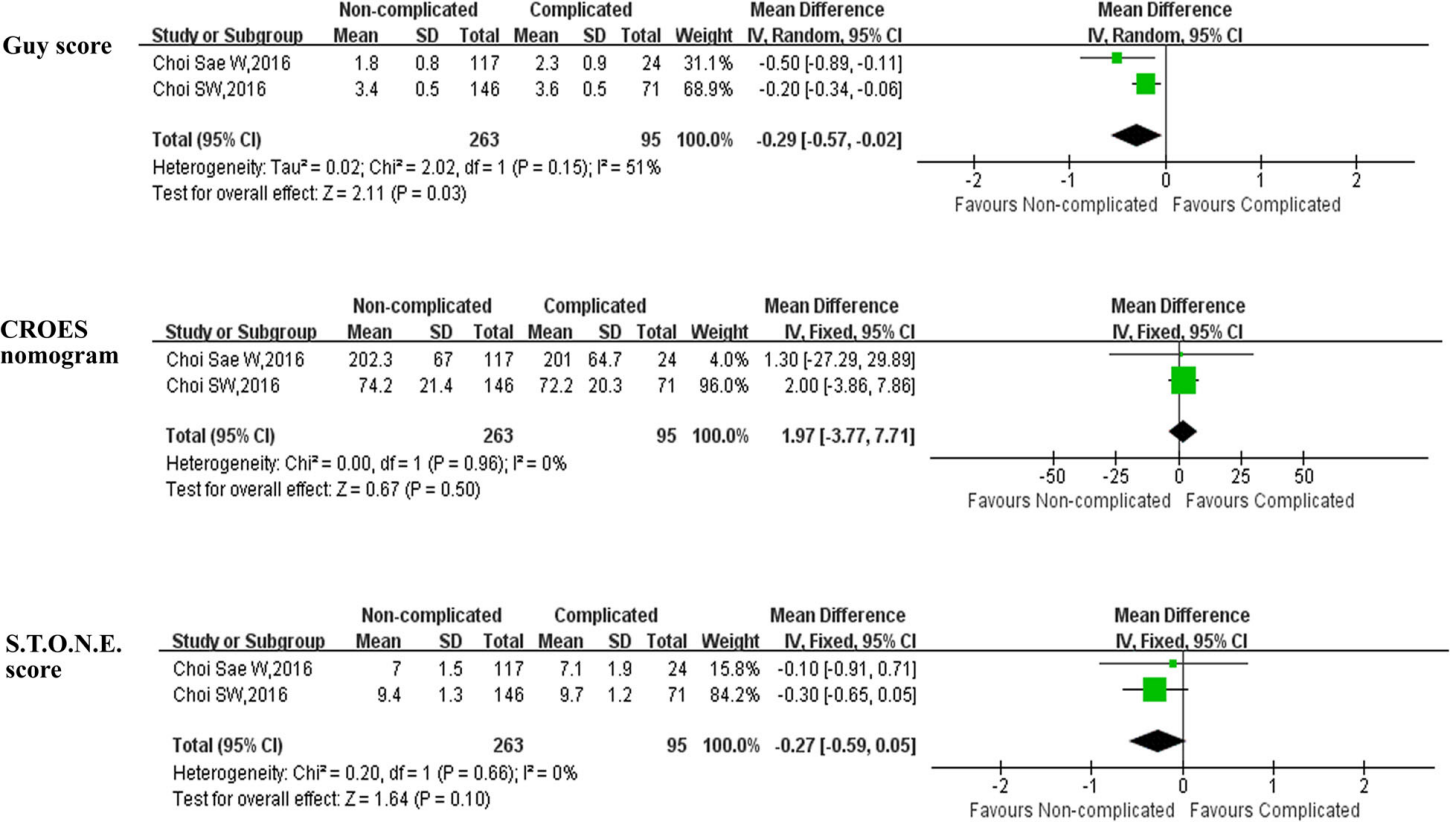
Forest plot and meta-analysis of outcomes of three scoring systems for predicting complications after PCNL

Pooled data of reported that the three SSSs in predicting post-PCNL complications, the forest plot indicated that Guy score was the only stone scoring system for predicting post-PCNL complications (WMD = -0.29, 95% CI: − 0.57 to − 0.02, *P* = 0.03) (Table 5, Fig. 5). No association between the two other scoring systems (CROES nomogram and S.T.O.N.E. score) and post-PCNL complications (Table 5, Fig. 5).

**Table 5:** Overall analysis of three scoring systems for predicting complications after PCNL

Outcome of interest	No. of studies	No.of patients Non-complicated/complicated	OR/WMD(95%CI) ^a^	*p*-value	Study heterogeneity			
					Chi^2^	df	*I* ^*2*^	*p*-value
Guy score	2	263/95	−0.29[− 0.57,-0.02]	**0.03**	2.02	1	51%	0.15
CROES nomogram	2	263/95	1.97[−3.77,7.71]	0.50	0.00	1	0%	0.96
S.T.O.N.E. score	2	263/95	−0.27[−0.59,0.05]	0.10	0.20	1	0%	0.66

*PCNL* percutaneous nephrolithotomy, *CROES* clinical research office of the endourological society scoring system, *Guy* Guy scoring system, *S.T.O.N.E* S.T.O.N.E scoring system, *OR* odds ratio, *WMD* weighted mean difference, *CI* confidence interval, ^a^ OR

## Discussion

Widely applicable and straightforward tools will highly improve patient counseling, clinical decision making, assessment of operation outcomes and academic study after PCNL for renal stones [22, 23]. These can allow reliable and accurate comparisons of treatment safety and efficacy, and facilitate the meaningful comparison of clinical studies [24]. We considered that the commonest and validated SSSs (Guy score, CROES nomogram, and S.T.O.N.E. score) could be predictive of SFR and complications after PCNL.

Both similar and divergent variables between the three SSSs has to be known. The Guy stone score consists of four grades based on stone burden and patient anatomy [7, 25]. The CROES nomogram is highly generalizable based on global data and uniquely grades risk across a continuous scale rather than dividing stones of varying complexity into discrete groups [7, 25]. The S.T.O.N.E. stone score stratifies patients into low-, moderate-, and high-risk groups, and it is more useful for decision making [7, 25]. The three SSSs included different parameters, however, the stone location, stone count and staghorn calculi were pivotal variables in the three SSSs [7].

On the other hand, Guy stone score included renal anatomy but not stone burden, and this was a difference from CROES nomogram and S.T.O.N.E. score. The CROES nomogram included prior treatment as well as operation volume, and these variables had remarkable relationships to the SFR. However, CROES nomogram lacked imageology information on hydronephrosis and calyceal abnormalities. The S.T.O.N.E. score comprised stone size, tract length, obstruction, number of involved calices and essence, and it had greater feasibility and accuracy than any of the individual variables alone. In our meta-analysis, the results showed preoperative variables gender, stone burden, number of involved calyces and staghorn calculi were remarkably correlating with SFR after PCNL for kidney calculi.

However, there is still no widely accepted stone scoring system for the prediction of outcomes after PCNL, and contradictions between different authors exist concerning the prediction of outcomes by the SSSs. Some experts reported that three SSSs were efficacy and equally predictive of SFR by estimating and comparing the three SSSs in 246 patients after PCNL [17]. Tailly et al. reported that three SSSs have similar predictive accuracy of SFS by comparing the three SSSs in 586 patients after PCNL, but no association between three SSSs and complications [20]. Moreover, Bozkurt et al. reported that both the Guy and CROES nomogram had a remarkable relationship with SFS and complications [12]. Noureldin et al. also reported that the Guy score and S.T.O.N.E. score were equally associated with SFS, but there were no significant correlations with complications [18]. However, when other experts evaluated and compared three SSSs in predicting post-PCNL SFR and complications, the results showed that only S.T.O.N.E. score was a predictor of SFS after PCNL for renal stones [13]. Kocaaslan et al. reported that the CROES nomogram was well correlated with the success of PCNL in cases with anatomical abnormalities, and that the Guy score and S.T.O.N.E. score failed to predict the SFS and complications after PCNL [16]. Choi et al. compared the predictability and accuracy of the Guy score, CROES nomogram and S.T.O.N.E. score, the results showed that only Guy score could predict SFS and complications after PCNL [14]. Sfoungaristos et al. also compared three SSSs, and the results indicated that S.T.O.N.E. score was the only predictor of the SFR for post-PCNL, and the three SSSs were not associated with complications [19]. In our meta-analysis, the results indicated that the three SSSs were remarkably associated with SFS and equally predictive of SFR, but only Guy score was a predictor of complications.

However, some critical limitations exist in the three SSSs. Firstly, stone burden and density as the important parameters dide not reflect in Guy’s score. Moreover, it is also failed to describe procedure difficulties as well as clinical variability. Secondly, the CROES nomogram is also did not reflect stone density and lacked important variables affecting the outcomes, including imaging information on hydronephrosis and pelvicalyceal abnormalities [5, 26]. Moreover, the CROES nomogram was complex in the clinical applications [17]. Thirdly, the limitations of S.T.O.N.E. score were validated with a small cohort which may limit its widespread usage [4, 7].

Similarly, several limitations existed while analyzing and interpreting results in our meta-analysis. Firstly, to identify prognostic factors, we acknowledge that other variables, such as surgeon experience and advanced surgical instruments, may need further investigation. These factors in the future may need to be incorporated in multicenter and larger samples clinical applications. Secondly, there existed heterogeneities of studies, some studies in this meta-analysis had the risk of selection bias. Lastly, all patients were evaluated for SFR after PCNL by KUB but not by CT, which may have overstated the SFR. Therefore, a need to develop more accurate and practical SSS to assess the relationship between the SSS and SFR, complications.In conjunction, our meta-analysis thus provides some up to date conclusions for the advantages and disadvantages of three SSSs in predicting of SFS and complications.

## Conclusions

The Guy score, CROES nomogram and S.T.O.N.E. score were equally accurate predictive of SFR in patients undergoing PCNL, but the Guy score is the only SSS for predicting complications.

## Additional file

Additional file 1: **Figure S1.** Forest plot and meta-analysis of demographic and clinical characteristics compared stone-free with not stone-free after PCNL. (TIF 3058 kb)

## Data Availability

All data generated or analyzed during this study are included in this published article.

## Abbreviations

AUC: Area under curves
CROES: The Clinical Research Office of the Endourological Society nomogram
EAU: European Association of Urology
FE: Fixed effects
LOE: Level of evidence
NOS: The Newcastle-Ottawa Scale
ORs: Odds ratios
PCNL: Percutaneous nephrolithotomy
RE: Random-effects
SFR: Stone free rate
SSS: Three stone scoring systems
the S.T.O.N.E: stone size, tract length, obstruction, number of involved calices and essence
WMDs: Weighted mean differences
