# Supplementary figures and images for: Evaluation of three stone-scoring systems for predicting SFR and complications after percutaneous nephrolithotomy: a systematic review and meta-analysis
Source: BMC Urol. 2019 Jul 1;19:57. doi: 10.1186/s12894-019-0488-y (PMC6604199; doi:10.1186/s12894-019-0488-y)

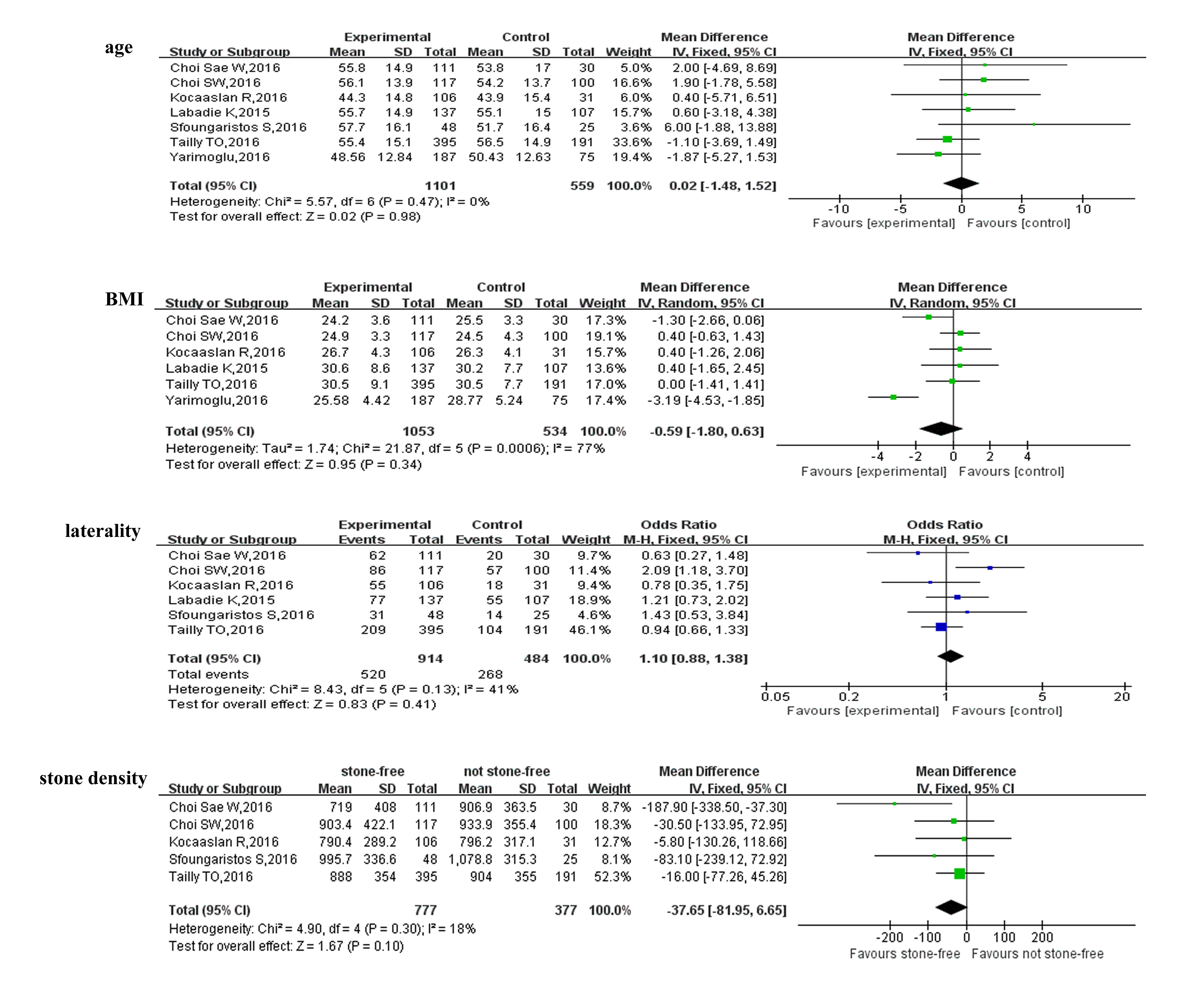

Supplement: Supplementary file 1 — Figure S1. Forest plot and meta-analysis of demographic and clinical characteristics compared stone-free with not stone-free after PCNL. (TIF 3058 kb) [file 12894_2019_488_MOESM1_ESM.tif]
